# Correlation of CCL8 expression with immune cell infiltration of skin cutaneous melanoma: potential as a prognostic indicator and therapeutic pathway

**DOI:** 10.1186/s12935-021-02350-8

**Published:** 2021-11-29

**Authors:** Peipei Yang, Wanrong Chen, Hua Xu, Junhan Yang, Jinghang Jiang, Yunhui Jiang, Ganglin Xu

**Affiliations:** 1 Department of Dermatology, Jingmen No. 2 People’s Hospital, No. 39 Xiangshan Road Dongbao Zone, Jingmen, 448000 Hubei China; 2 Graduate School, Guangxi Medical University, Nanning, Guangxi Zhuang Autonomous Region China; 3 Department of Pathology, Jingmen No. 2 People’s Hospital, No. 39 Xiangshan Road Dongbao Zone, Jingmen, 448000 Hubei China; 4 The Reproductive Medicine Center, Jingmen No. 2 People’s Hospital, Jingmen, Hubei China

**Keywords:** Tumor microenvironment, Tumor-infiltrating immune cells, Skin cutaneous melanoma, M1 macrophages

## Abstract

**Background:**

The tumor microenvironment (TME) is critical in the progression and metastasis of skin cutaneous melanoma (SKCM). Differences in tumor-infiltrating immune cells (TICs) and their gene expression have been linked to cancer prognosis. Given that immunotherapy can be effective against SKCM, we aimed to identify key genes that regulate the immunological state of the TME in SKCM.

**Methods:**

Data from 471 SKCM patients in the The Cancer Genome Atlas were analyzed using ESTIMATE algorithms to generate an ImmuneScore, StromalScore, and EstimateScore for each patient. Patients were classified into low- or high-score groups based on median values, then compared in order to identify differentially expressed genes (DEGs). Then a protein–protein interaction (PPI) network was developed, and a prognostic model was created using uni- and multivariate Cox regression as well as the least absolute shrinkage and selection operator (LASSO). Key DEGs were identified using the web-based tool GEPIA. Profiles of TIC subpopulations in each patient were analyzed using CIBORSORT, and possible correlations between key DEG expression and TICs were explored. Levels of CCL8 were determined in SKCM and normal skin tissue using immunohistochemistry.

**Results:**

Two scores correlated positively with the prognosis of SKCM patients. Comparison of the low- and high-score groups revealed 1684 up-regulated and 18 down-regulated DEGs, all of which were enriched in immune-related functions. The prognostic model identified CCL8 as a key gene, which CIBERSORT found to correlate with M1 macrophages. Immunohistochemistry revealed strong expression in SKCM tissue, but failed to detect the protein in normal skin tissue.

**Conclusions:**

CCL8 is a potential prognostic marker for SKCM, and it may become an effective target for melanoma in which M1 macrophages play an important role.

## Background

Skin cutaneous melanoma (SKCM) is a common potentially malignant condition, and its prevalence has increased more than sixfold in the last six decades [1]. In 2020, more than 324,000 new SKCM cases and 57,000 deaths occurred, representing about one in 19 cancer cases and 23 cancer deaths [2, 3]. The annual incidence of SKCM will likely remain high as populations increase.

The distribution of melanoma subtypes varies with geography and ethnicity: in China, acral and mucosal SKCM are the most frequent subtypes of melanoma [4, 5]. Surgical resection is the standard treatment for early-stage SKCM, when it is often curative [6]. Systemic therapy is the standard for advanced SKCM, which is associated with poor prognosis [7, 8].

SKCM is associated with abnormal regulation of tumor-related genes [7, 9–11], including genes related to immune responses [12]. Extracellular matrix growth factors, secreted metabolites, and dynamic interactions among endothelial, interstitial, and immune cells create a tumor microenvironment (TME) that strongly affects tumor proliferation, differentiation, migration, and invasion [13–15]. In this way, mesenchymal cells, tumor-infiltrating immune cells (TICs), and fibroblasts regulate tumor progression [16–20], while tumor-associated macrophages infiltrate the TME, stimulating SKCM angiogenesis, growth and invasion [21, 22]. Thus, immunotherapy is increasingly being used in cancer treatment [7, 9, 10, 23, 24], but it can be rendered ineffective because of the TME and tumor heterogeneity. In this way, TICs in the TME are a key determinant of the therapeutic response.

Therefore, it is important to identify biomarkers that may facilitate early identification of SKCM or help predict response to treatment. Toward that end, we used the ESTIMATE algorithm to characterize the TME, and we established a prognostic model using Cox regression and the Least Absolute Shrinkage and Selection (LASSO). The model identified four differentially expressed genes (DEGs), one of which, CCL8, was identified as key by the web-based tool GEPIA. Expression of this gene was found to correlate with tumor infiltration by M1 macrophages using the CIBERSORT algorithm.

## Materials and methods

### Samples

Transcriptome data for SKCM tissues and adjacent non-cancerous tissues from 471 patients were extracted from The Cancer Genome Atlas (https://portal.gdc.cancer.gov/), together with the patients’ clinical information. Tissues were also obtained from SKCM patients at our institution.

This study was approved by the Ethics Committee of Jingmen No. 2 People’s Hospital, and procedures involving humans and their samples were performed in accordance with the Declaration of Helsinki.

### Calculation of immune and stromal scores using the ESTIMATE algorithm

We used the “ESTIMATE” algorithm in R (https://www.r-project.org/) to determine an EstimateScore, ImmuneScore, and StromalScore for the TME of SKCM tissue based on transcriptomic data. For each score, patients were assigned to low- or high-score groups according to the median value for that score. Survival of the two groups was compared using the “survminer” package in R, and potential correlations between scores and clinical characteristics were explored.

### DEGs and their functional analysis

Within each of the three scores, we compared the low- and high-score groups using the “limma” package in R in order to identify DEGs between them. DEGs with a |log_2_(fold change)|> 1 and false discovery rate < 0.05 were considered significant and retained in the analysis. Heatmaps of DEGs were created using the “pheatmap” package in R.

Potential functions of DEGs were identified by examining them for enrichment in Gene Ontology (GO) terms and Kyoto Encyclopedia of Genes and Genomes (KEGG) pathways. Enrichment was considered significant if *P* < 0.05 and *q* < 0.05.

### Protein–protein interaction (PPI) network

PPI networks of DEGs were constructed using the STRING website (string-db.org) at a confidence level of 0.9. The 30 genes with the largest numbers of interconnected nodes were visualized using the “cytoHubba” plug-in in Cytoscape software.

### Prognostic model

A total of 454 SKCM patients in The Cancer Genome Atlas, for whom complete survival data were available, were randomly divided 1:1 between a training group and a validation group. In the training group, patients were divided into low- and high-score groups based on median risk score, and their Kaplan–Meier survival curves were compared. Univariate Cox regression was used to identify immune-related genes that were significantly related to overall survival of SKCM patients based on the corresponding hazard ratio. A de novo prognostic model was created using LASSO and the “glmnet” package in R, then the genes in the model were refined using multivariate Cox regression.

The final prognostic model was validated by calculating a consistency index, calibration chart, and areas under the receiver operating characteristic curve (AUCs).

In addition, we validated the differential expression of model genes between normal and tumor tissues using GEPIA (http://gepia.cancer-pku.cn/), and we examined potential correlations between the key genes obtained and patients’ clinical features.

These analyses involved the following packages in R: “rms”, “foreign”, “survival”, “survivalROC”, and "stdca.R". Results were considered significant if associated with P < 0.05.

### Analysis of TICs

The CIBERSORT algorithm was used to determine relative proportions of 22 types of immune cells in the SKCM patients from The Cancer Genome Atlas. After filtering out data that did not meet the significance criterion of *P* < 0.05, 228 patients were retained in the final analysis.

Based on median levels of immune cell content, we stratified patients into low- or high-level groups and compared their survival using the Kaplan–Meier method in order to identify immune cell types associated with survival. We also explored the correlation between key genes and the 22 types of immune cells.

### Immunohistochemistry of CCL8

SKCM and normal tissues were collected from surgical samples of patients treated at Jingmen No. 2 People’s Hospital. The tissue was paraffin-embedded and the paraffin blocks were cut into 4-μm sections, dewaxed in xylene, and hydrated through a graded series of ethanol solutions (100, 90, 80, and 70%). The sections were incubated in 1 × ethylenediaminetetraacetic acid antigen repair solution for 20 min at room temperature, then washed three times with tap water. Endogenous peroxidases were blocked by soaking sections in 3% H_2_O_2_ for 10–30 min.

Sections were stained with primary rabbit antibody against CCL8 (catalog no. NBP1-79937, NOVUS Biologicals, USA) at a dilution of 1:100 at 4 °C. The sections were washed three times with phosphate-buffered saline (PBS) for 5 min, incubated with anti-rabbit secondary antibody at room temperature for 10 min, rinsed again in PBS, and incubated with anti-horseradish peroxidase conjugate for 10 min. Finally, sections were counterstained with hematoxylin for 1–3 min, rinsed three times with PBS, and incubated in 1% hydrochloric acid-alcohol. Two expert pathologists independently analyzed the sections.

## Results

### Correlations between scores and survival of SKCM patients

**Fig. 1:**
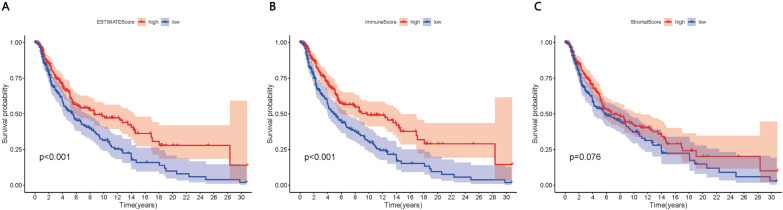
Correlation of **A** EstimateScore, **B** ImmuneScore and **C** StromalScore with overall survival of SKCM patients, based on Kaplan–Meier analysis

Kaplan–Meier analysis was used to explore correlations of the EstimateScore, ImmuneScore and StromalScore with survival of SKCM patients (Fig. 1A–C). The first two scores correlated significantly with prognosis (P < 0.001).

### Correlations between scores and clinical features of SKCM patients

**Fig. 2:**
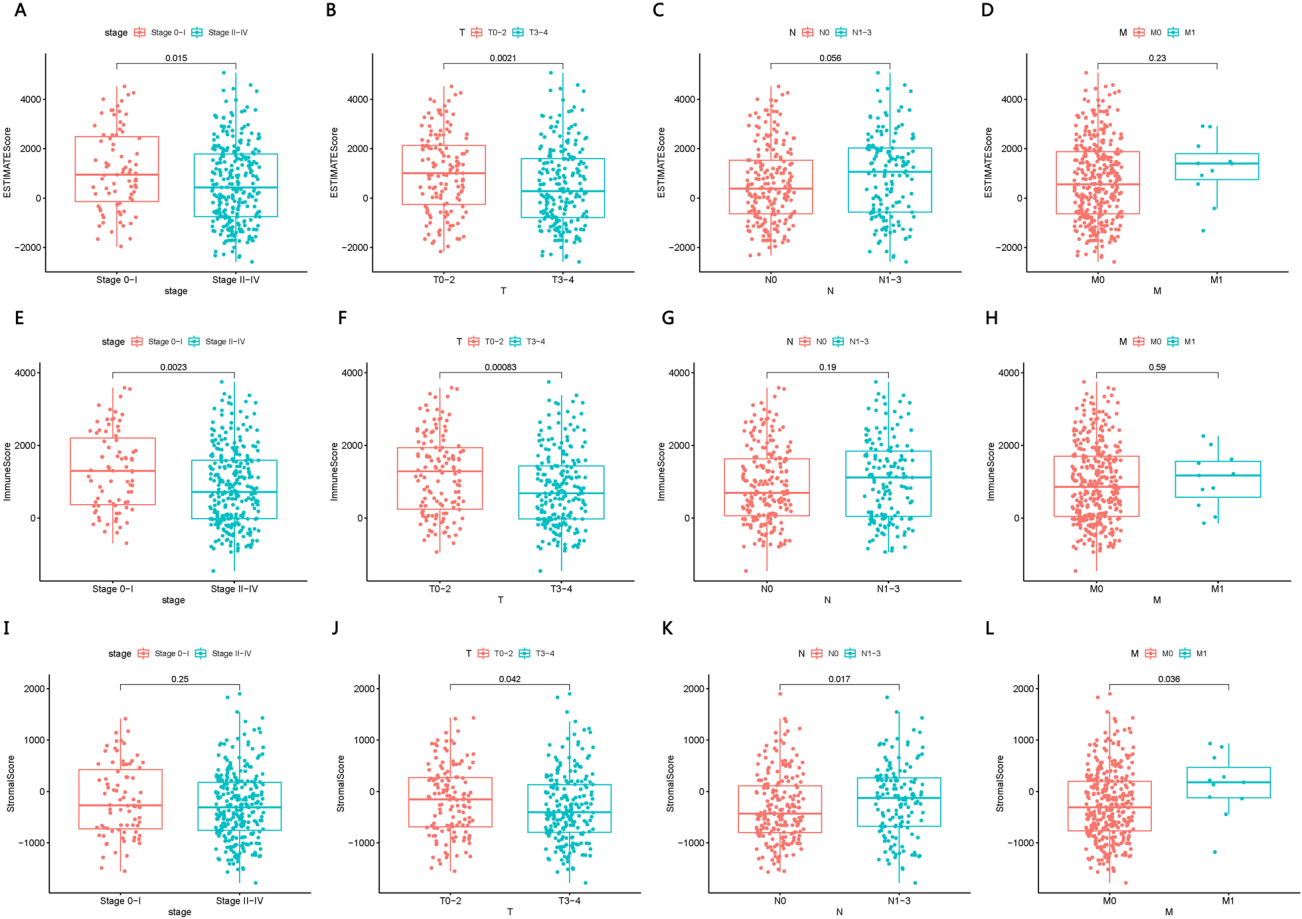
Correlation of **A**–**D** EstimateScore, **E**–**H** ImmuneScore and **I**–**L** StromalScore with clinical features of SKCM patients

EstimateScores and ImmuneScores, but not StromalScores, were significantly different between patients in stage 0-I or stage II-IV (Fig. 2A–L). All three scores differed significantly between patients in stage T0-2 or T3-4. StromalScores, but not the other two scores, differed significantly between patients in stage N0 or N1-3, as well as between patients in stage M0 or M1.

### DEGs between patients with low or high scores

**Fig. 3:**
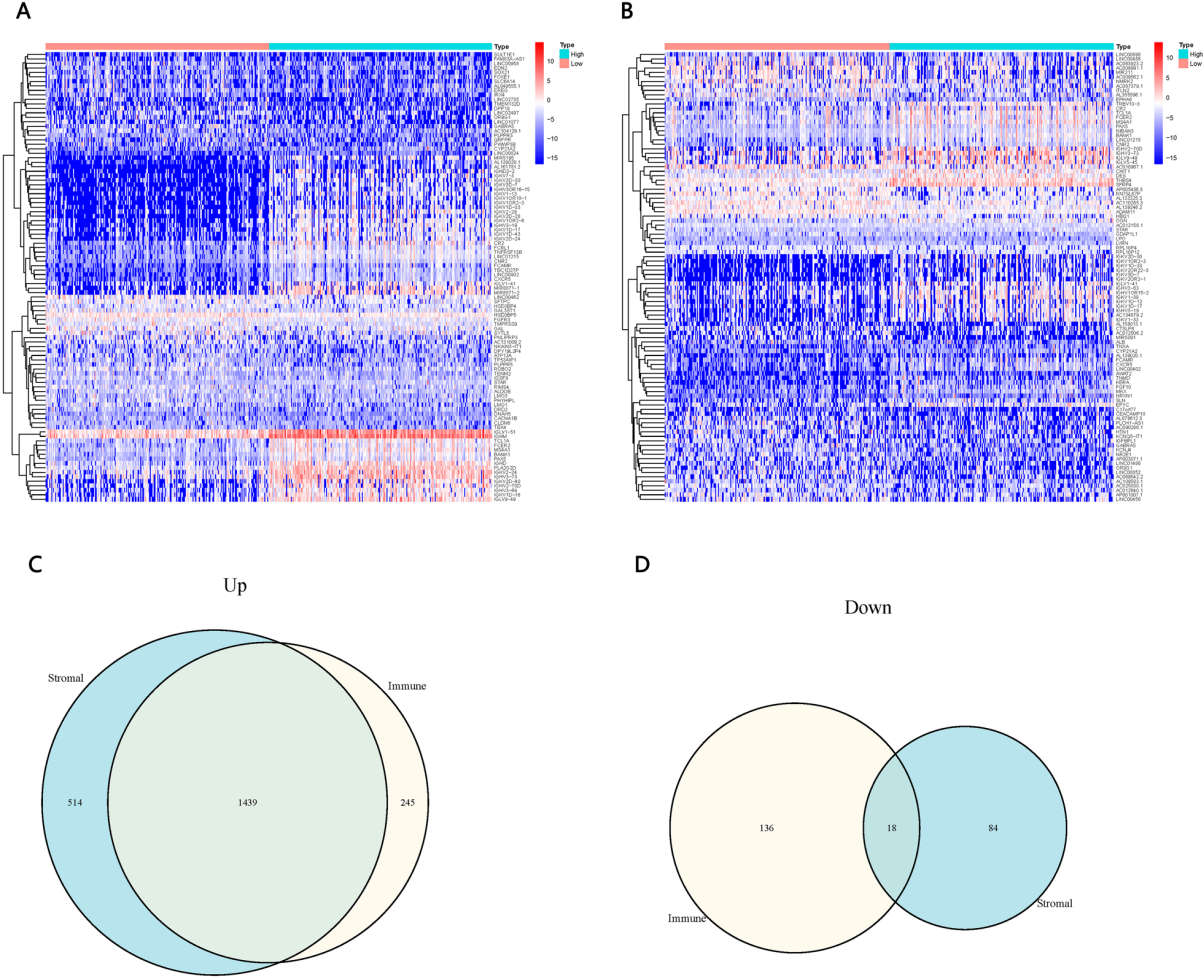
Differentially expressed genes (DEGs) between patients with low or high ImmuneScores or StromalScores. **A**, **B** Heat map of the top 50 up- and down-regulated DEGs between patients with low or high scores. **C**, **D** Venn plot showing the DEGs common to the comparisons based on ImmuneScore or StromalScore

We identified 1684 up-regulated genes and 154 down-regulated DEGs between patients with low or high ImmuneScores (Fig. 3A). We also identified 1953 up-regulated and 102 down-regulated DEGs between patients with low or high StromalScores (Fig. 3B). A total of 1684 up-regulated and 18 down-regulated genes overlapped between the two sets of DEGs (Fig. 3C, D).

### Functional enrichment of DEGs

**Fig. 4:**
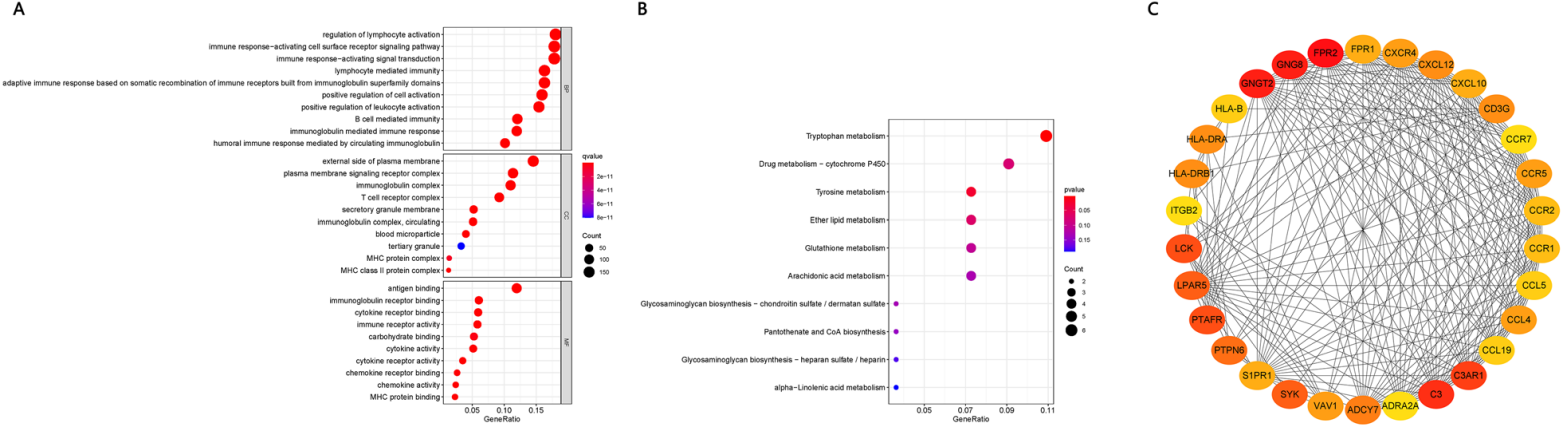
Functional Enrichment of DEGs. **A** Enrichment in Gene Ontology (GO) terms. **B** Enrichment in Kyoto Encyclopedia of Genes and Genomes (KEGG) pathways. **C** Protein–protein interaction (PPI) network for the 30 genes with the largest numbers of interconnected nodes

DEGs were enriched mainly in the GO terms related to immunological response, immunoreceptor components, and immunoglobulin receptor binding (Fig. 4A). DEGs were enriched mostly in the KEGG pathways related to synthetic and metabolic pathways (Fig. 4B). The 30 genes with the largest numbers of interconnected nodes were calculated and displayed in the PPI network (Fig. 4C).

### Hub gene identification and establishment of a prognostic model

**Fig. 5:**
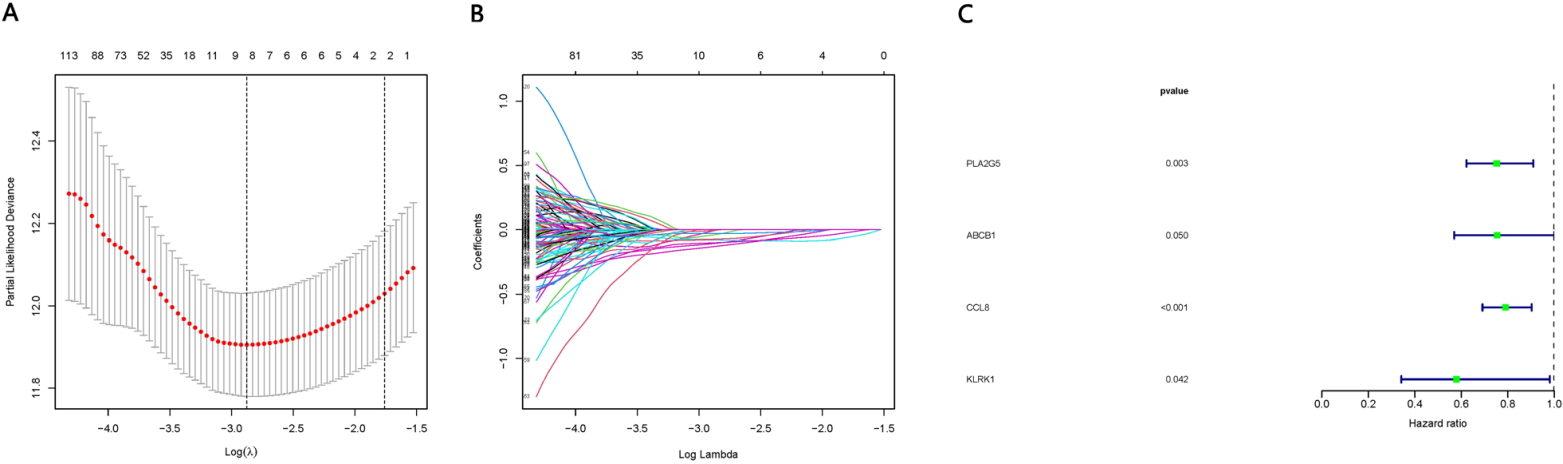
Establishment of a prognostic model. **A** Cross-validation to select the tuning parameter for the least absolute shrinkage and selection operator (LASSO) model for overall survival. **B** LASSO coefficients for DEGs associated with overall survival of SKCM patients. **C** Forest plots of the four genes in the final prediction model (PLA2G5, ABCB1, CCL8, and KLRK1), based on multivariate Cox regression

Significant DEGs were selected for LASSO regression after single-factor Cox regression analysis, and nine genes were filtered and screened for multi-factor Cox regression. Multi-factor Cox regression identified four genes closely related to overall survival of SKCM patients: PLA2G5, ABCB1, CCL8, and KLRK1 (Fig. 5).

### Validation of the prognostic model

**Fig. 6:**
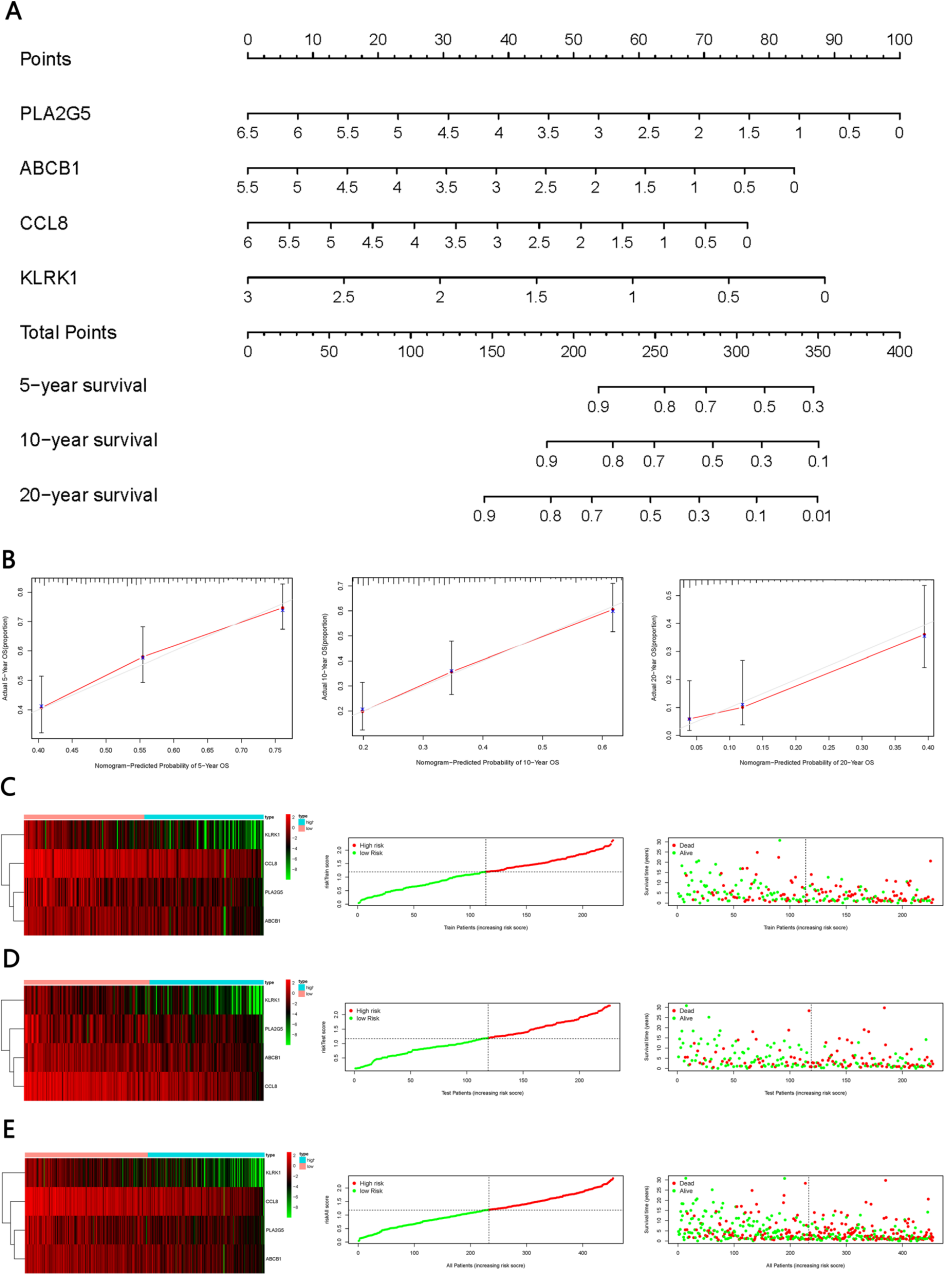
Validation of the prognostic model. **A** Nomogram for predicting overall survival of SKCM patients at 5, 10 and 20 years, based on the four key genes. **B** Calibration curves for predicting patient survival at 5, 10 and 20 years. **C** Heat map showing expression of the four genes among patients in the training group, stratified by overall survival. Distribution of the four-gene risk score and survival times of patients in the training group. **D** Heat map showing expression of the four genes among patients in the validation group, stratified by overall survival. Distribution of the four-gene risk score and survival times of patients in the validation group. **E** Heat map showing expression of the four genes among all patients in study, stratified by overall survival. Distribution of the four-gene risk score and survival times of all patients

**Fig. 7:**
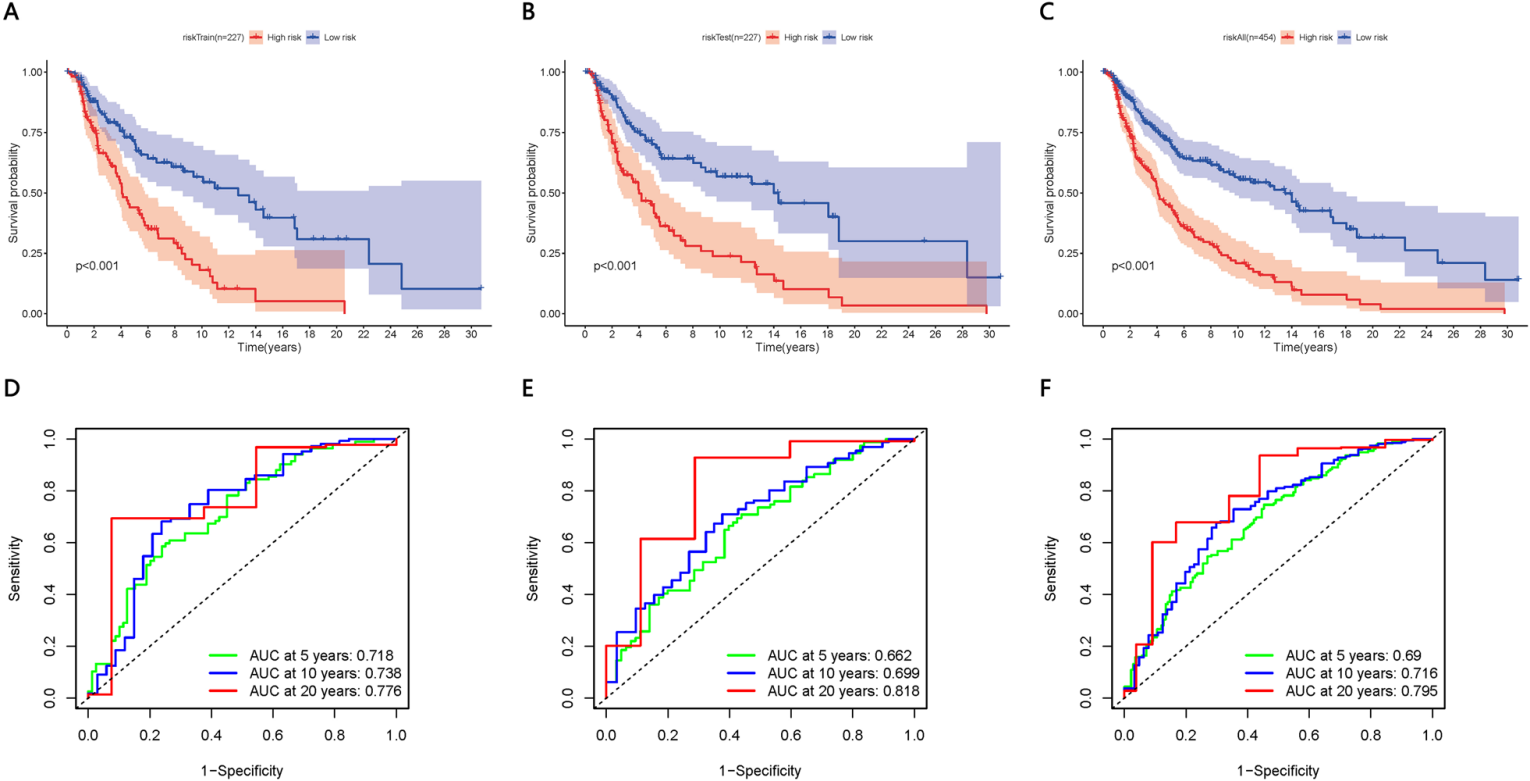
The four-gene signature risk score was determined based on Kaplan–Meier analysis of overall survival and on receiver operating characteristic curves. **A**–**C** Analysis of the training group, validation group and all patients in the study. **D**–**F** Time-dependent receiver operating characteristic curves for 5, 10 and 20 years in the training group, validation group, and all patients in the study

We constructed a prognostic model based on the abovementioned four genes, as well as a nomogram for predicting overall survival at 5, 10 and 20 years (Fig. 6A, B). In the training group, Kaplan–Meier analysis showed lower survival rates among patients in the high-risk group than among patients in the low-risk group. High-risk patients showed low expression of all four genes (Fig. 6C, E). Similar results were obtained in the validation dataset and in the dataset comprising all patients (Fig. 7A–C). AUCs at 5, 10, and 20 years were as follows: training group, 0.718, 0.738, and 0.776 (Fig. 7D); validation group, 0.662, 0.699, and 0.818 (Fig. 7E); and all patients, 0.69, 0.716, and 0.795 (Fig. 7F).

**Fig. 8:**
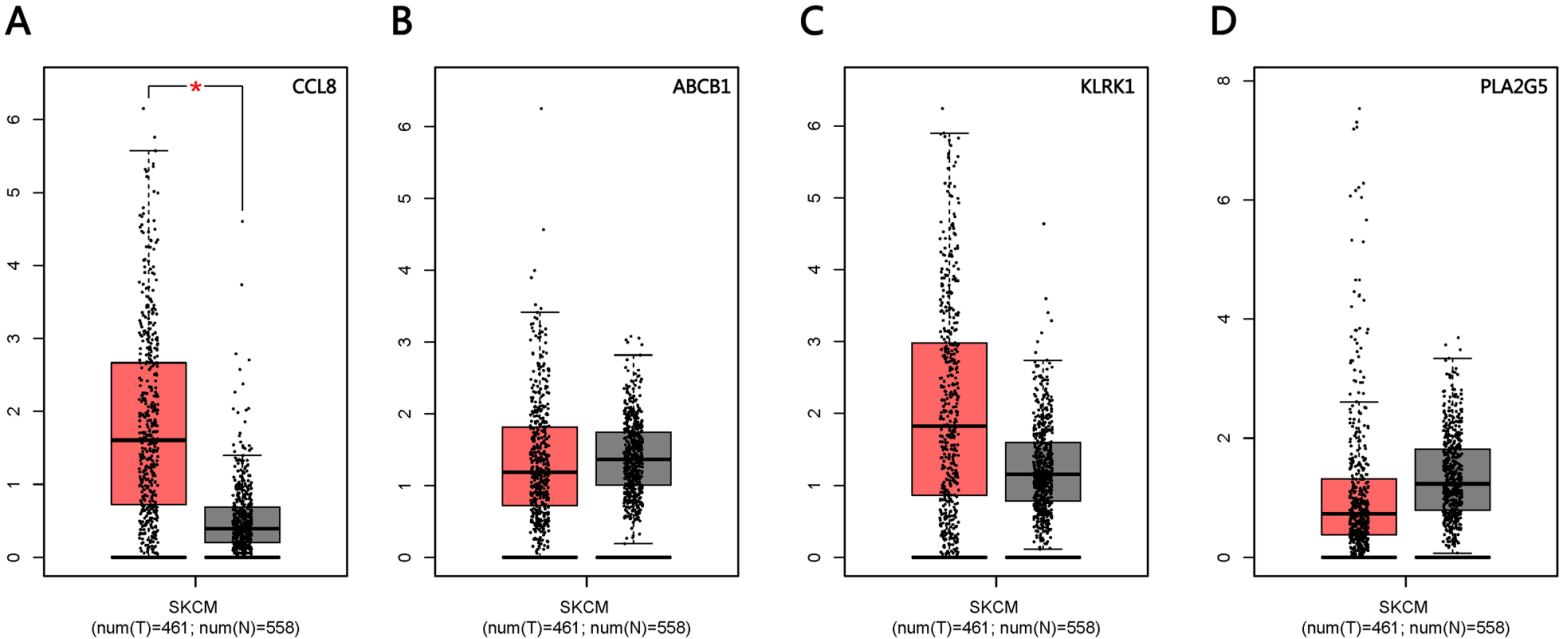
Verification of survival differences based on GEPIA. **A**–**D** Differences in the expression of CCL8, ABCB1, KLRK1, and PLA2G5 between tumor and normal tissues. The difference in CCL8 expression was statistically significant

**Fig. 9:**
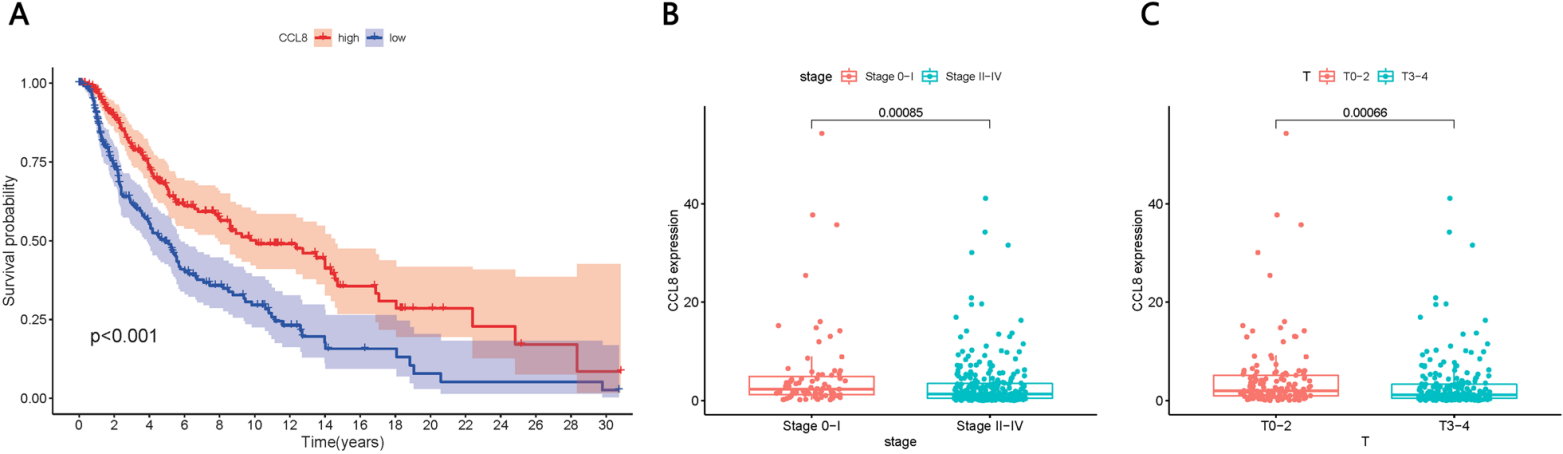
The prognostic role of CCL8 in SKCM. **A** Relationship between CCL8 expression and SKCM prognosis. **B**, **C** Correlation between CCL8 expression and clinical features

Using GEPIA, we verified the survival differences between patients expressing low or high levels of the four genes, and we found significantly higher CCL8 expression in tumors (Fig. 8A–D). Stratifying patients based on median expression of CCL8 showed that those expressing high levels of the protein had significantly longer overall survival (P < 0.001; Fig. 9A). In addition, CCL8 expression differed significantly between patients in stage II–IV or stage 0–I (Fig. 9B), as well as between patients in stage T0-2 or T3-4 (Fig. 9C).

### CCL8 expression and TICs

**Fig. 10:**
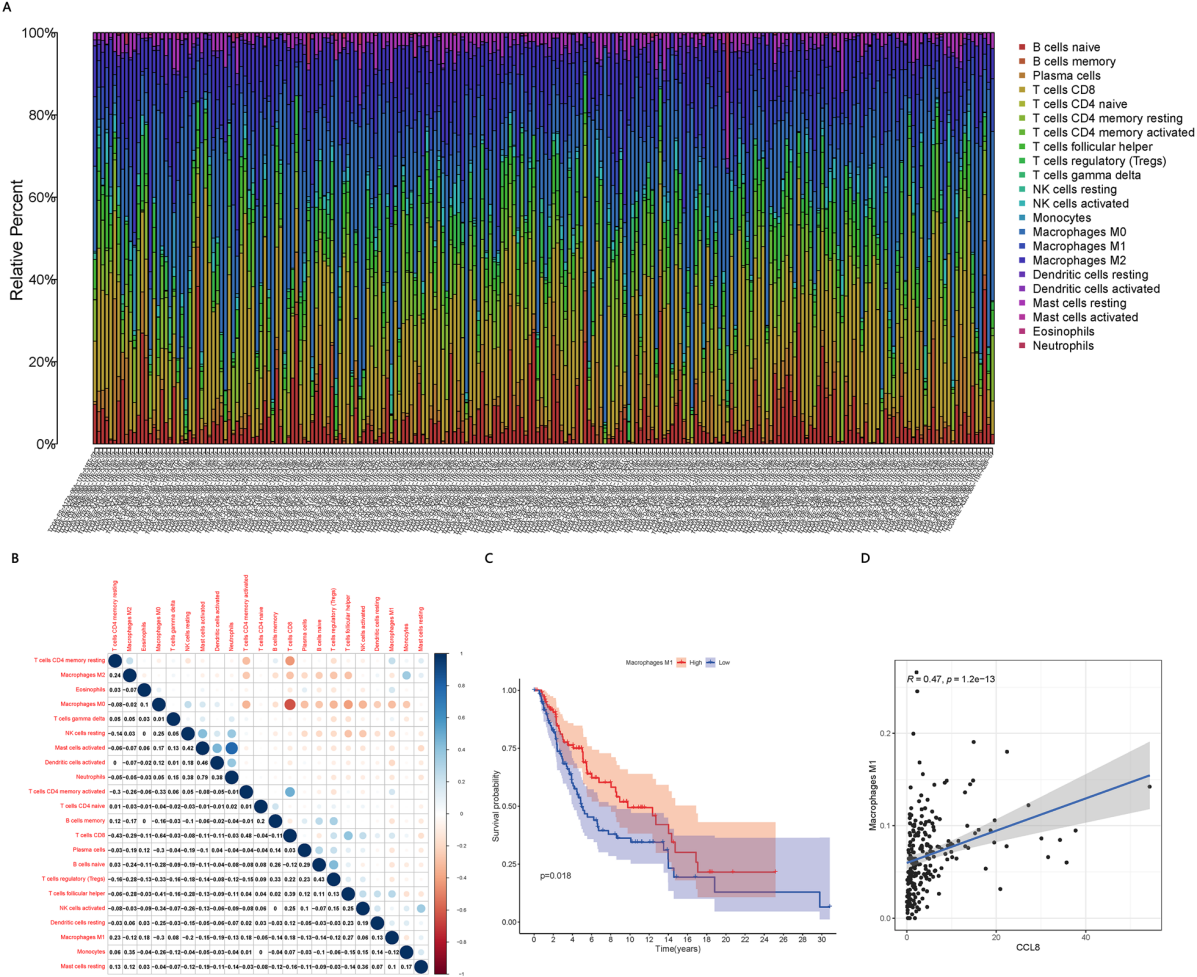
Analysis of TICs and CCL8. **A** Relative proportions of 22 immune cell types in each patient, based on the CIBERSORT algorithm. **B** Correlations among the 22 immune cell types. Blue and red represent positive and negative relationships, respectively. **C** Correlation between M1 macrophages and patient survival. **D** Positive correlation between CCL8 expression and M1 macrophages

Among the 228 patients for which CIBERSORT analysis of TICs passed the significance threshold (Fig. 10A, B), patients showing high levels of M1 macrophages showed better survival than patients with low levels (Fig. 10C). In addition, CCL8 expression correlated positively with M1 macrophages (Fig. 10D).

### Immunohistochemistry of CCL8

**Fig. 11:**
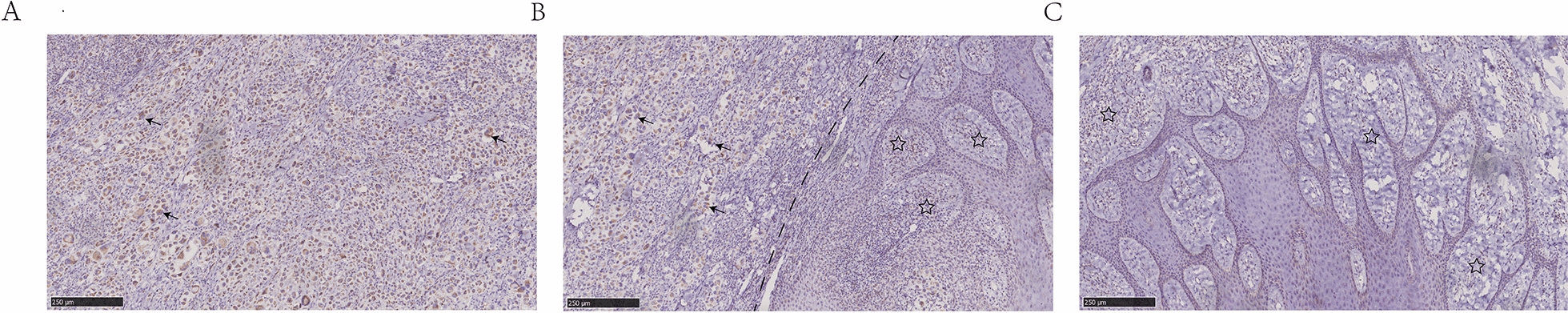
Immunohistochemical analysis of CCL8. **A** Positive immunostaining for CCL8 in the cytoplasm of SKCM tissue. **B** Positive immunostaining is visible in SKCM cells (*left*) but not in adjacent normal cells (*right*) within the same surgical sample. **C** CCL8 was undetectable in normal skin tissue. Arrow, anti-CCL8 staining; pentagon, lack of anti-CCL8 staining

To verify our in silico analyses, we investigated CCL8 expression in SKCM and normal skin tissues. SKCM tissue showed abundant cytoplasmic CCL8 expression (Fig. 11A), whereas the protein was undetectable in adjacent normal tissue (Fig. 11B, C). Clinical features of the patients are shown in Table 1.

**Table 1:** Clinical features of the six SKCM patients whose tissues were analyzed by immunohistochemistry

Patient no.	Sex	Age, year	Tumor size, cm
1	Female	42	2.1 × 1.4 × 0.7
2	Male	38	1.9 × 1.3 × 0.5
3	Male	57	3.2 × 1.9 × 0.8
4	Female	63	1.5 × 1.0 × 1.0
5	Male	38	2.8 × 2.1 × 1.3
6	Female	50	2.7 × 1.6 × 0.5

## Discussion

Only 5% of all cases of skin cancer are SKCM and its global incidence is estimated to be 15–25 per 100,000 individuals, yet it accounts for 80% of fatal cases of skin cancer [25, 26]. If metastasis occurs, SKCM can lead to intractable metastatic melanoma. The present study focused on TICs in the disease because researchers have increasingly been exploring possibilities of immunotherapy against SKCM [27, 28] and have achieved important successes [29, 30]. Immune cells in the TME interact with tumor cells and other cell types to strongly influence cancer progression [31]. Therefore, finding tumor biomarkers that may be linked to the SKCM and prognosis is essential.

Our analysis of immune and stromal components in the TME of SKCM using the ESTIMATE algorithm identified four putative prognostic biomarkers, one of which we verified experimentally: CCL8. Also known as monocyte chemotactic protein-2CC, CCL8 is one of the smallest chemokines and it triggers chemotactic activity in monocytes, lymphocytes, basophils, and eosinophils [26, 32]. Previous work has indicated an oncogenic role for CCL8: its expression correlates positively with progression of various cancers [33, 34], including breast cancer [35, 36], and with metastasis of cancers to the lung [37]. Conversely, the present study suggests that, at least in melanoma, CCL8 can play a protective role: high levels of CCL8 correlated with better prognosis. Our findings are consistent with a report that combining CCL8 with CCR5 can treat rectal cancer [38].

CIBERSORT analysis of SKCM patients in our study showed that a higher proportion of M1 macrophages among TICs was associated with better survival. M1 macrophages, also known as classically activated macrophages, secrete pro-inflammatory factors to support immune responses. In fact, M1 macrophages can be exploited to deliver antitumor drugs [39–41], and therapy that simultaneously upregulates M1 macrophages and downregulates M2 macrophages can be effective against cancers [42]. Our study found a positive correlation between CCL8 expression and levels of M1 macrophages, suggesting that CCL8 may help drive the anti-tumor efficacy of M1 macrophages in the TME. Indeed, one study found that CCL8 sensitized mice with cutaneous squamous cell carcinoma to photodynamic therapy by recruiting M1 macrophages [43]. In this way, our analyses suggest that CCL8 may influence immune activity in the TME, such as by promoting antitumor responses of M1 macrophages. This may help explain why CCL8 expression correlates with overall survival of SKCM patients.

## Conclusion

Based on bioinformatics analysis of The Cancer Genome Atlas, our study identifies CCL8 as a potential prognostic marker in SKCM and as part of a therapeutic pathway involving M1 macrophages that may be exploited to improve treatment options for patients.

## Data Availability

The public datasets used in our work can be found on https://portal.gdc.cancer.gov/ and http://gepia.cancer-pku.cn/. Immunohistochemical datasets used and/or analyzed during the current study are available from the corresponding author on reasonable request.
